# Determinants of postpartum long-acting reversible contraceptives in the extended postpartum period in Shashago district, Central Ethiopia: a cross-sectional study conducted in the community

**DOI:** 10.1186/s40834-024-00284-w

**Published:** 2024-05-10

**Authors:** Tesfaye Eristu, Abera Mekis, Ritbano Ahmed Abdo

**Affiliations:** 1 Department of Public Health, College of Medicine and Health Sciences, Wachemo University, Hossana, Ethiopia; 2 Department of Midwifery, College of Medicine and Health Sciences, Wachemo University, Hossana, Ethiopia

**Keywords:** Postpartum, Family planning, Long-acting contraceptives, Associated factors

## Abstract

**Background:**

Women who fail to initiate contraceptive use within the first year after childbirth face an increased likelihood of experiencing unintended pregnancies in close succession. In regions with limited resources, the use of postpartum contraceptives, particularly long-acting reversible contraceptives, remains notably low. Consequently, this study sought to assess the prevalence and determinants of postpartum long acting reversible contraceptives in the extended postpartum period in the Shashago district, Central Ethiopia.

**Methods:**

This study employed a community-based cross-sectional design, conducted between March 1, 2021, and April 15, 2021, involving a total of 617 women of reproductive age. The selection of study participants was performed using a multistage stratified sampling technique. Data collection was carried out through the use of a structured interviewer-administered questionnaire. Subsequently, the collected data were entered into Epi-data version 3.1 and exported to SPSS version 25 for further analysis. Bivariate and multivariable logistic regression analyses were conducted, and statistical significance was determined using a P value of 0.05, along with adjusted odds ratios (AORs) and their corresponding 95% confidence interval (CI).

**Results:**

A total of 224(36.3%) women used long-acting contraceptives after giving birth. Among these women, 31.1% used Implanon, while 5.2% used an intrauterine device (IUD). Factors significantly associated with the use of long-acting contraceptives after childbirth included age 25–29 years (AOR: 1.8, 95% CI: 1.1-3.0), age ≥ 35 years (AOR = 8.7, 95% CI: 3.6–21.5), primary education (AOR = 3.3, 95% CI: 1.6–6.7), secondary education and above (AOR = 3.5, 95% CI: 1.5–8.3), and history of abortion (AOR = 2.7, 95% CI: 1.3–5.4). Additionally, having good knowledge of long-acting contraceptives after childbirth (AOR: 2.4, 95% CI: 1.5–3.9) was significantly associated with their use.

**Conclusion:**

This study revealed that a small number of women opt for long-acting contraceptives after childbirth, with Implanon being more popular than IUDs. Factors such as age, education level, abortion history, pregnancy counseling, and knowledge about long-acting contraceptives were linked to their usage. Integrating contraceptive counseling into routine antenatal and postnatal care is essential for ensuring access to postpartum contraception. Tailored interventions based on age and education level could also help promote long-acting contraceptive use. More research and targeted interventions are needed to overcome these barriers and improve access to these methods for postpartum women.

## Introduction

Postpartum family planning involves preventing unintended pregnancies and closely spaced pregnancies within the first 12 months after childbirth. It can also extend up to two years after childbirth [1]. This intervention is important for women and their families to achieve the best health outcomes for both mothers and babies [2]. Pregnancy is a critical time that can impact a woman’s future health, so it is crucial to avoid complications [3–6]. The World Health Organization recommends at least a 24-month interval between births to ensure optimal health [7]. The use of contraception during the postpartum period helps women achieve healthy birth spacing and avoid pregnancies during times of increased risk [8–10].

Global efforts are underway to reduce maternal deaths by 2030, but progress needs to accelerate to reach Sustainable Development Goal 3.1. The current annual reduction in maternal mortality is only 2.1%, which is far below the target of 6.4%. Low-resource settings bear the highest burden of maternal deaths [11]. While Ethiopia has improved maternal health, it remains a concern, as in other low-income countries [12]. The initiation of contraceptives immediately after delivery prevents unintended pregnancy during the postpartum period, and women can be encouraged to continue the contraceptive method, which has long-term implications for their fertility intentions [13]. However, despite these advantages, the utilization of modern contraceptives during the postpartum period in low-income countries, including Ethiopia, is extremely low [14].

Long-acting reversible contraceptives (LARCs), defined as methods that are effective for at least 1 year, disrupt the linear event of pregnancy and childbearing and aim to help women attain their desired family size by delaying or avoiding childbearing during the use of the method, preventing unintended pregnancy and unwanted future childbearing [15–21]. Globally, 45.2% of women use long-acting family planning methods (female and male sterilization, IUD, implant), with variations across regions. Sub-Saharan Africa (SSA) and Europe rely more on injectables, while Asia, Latin America, and the Caribbean use implants and intrauterine devices more commonly [22]. Studies conducted in 26 SSA countries have shown that the prevalence of long-acting contraception is 21.7%, ranging from 1.9% in Namibia to 55.0% in Beni [23]. Unfortunately, the coverage of modern postpartum contraceptives, especially long-acting contraceptives, remains deficient in low-income countries [17–19, 24–28]. In SSA, the utilization of LARCs among postpartum women stands at only 12.6%, with significant variations observed between countries. These variations range from as low as 1.5% in Angola to as high as 19.5% in Senegal [29].

Several previous studies have been conducted to identify the factors associated with the adoption of LARCs in the postpartum period in various countries. The socioeconomic factors that have been found to influence uptake include residing in urban areas [30], having a high level of maternal education [19, 23, 31, 32], having a high wealth index [24, 29] and being employed [30, 32]. Reproductive factors include receiving family planning counseling during and immediately after antenatal care [19, 28, 31, 33], health facility child birth [29], planned birth [28], having a positive attitude towards LARC [33], resumption of sexual activity [31], attending antenatal care visits [29, 32], having five or more living children [23–25] and previous use of LARC [19, 20, 24]. Other facilitating factors for postpartum long-acting reversible contraceptive (PLARC) use include discussions about family planning with husbands or family members, proximity to health facilities, receiving respectful care during childbirth [33], and engaging in conversations about LARC with healthcare professionals [20]. Overall, studies examining the prevalence of PLARC uptake indicate significant variations depending on geographic location.

The Federal Ministry of Health of Ethiopia (FMOH) is committed to improving maternal and child health services in Ethiopia. This commitment is demonstrated through various initiatives and policies, such as the launch of the National Reproductive Health Strategy in 2006 and the training of health workers in essential and emergency obstetric care [34, 35]. The FMOH has demonstrated a commitment to improving maternal and infant health through various initiatives and policies. One of their policies is the Care for Newborn and Child Health Week campaign, which aims to provide essential health services for mothers and newborns [35]. Additionally, the Ministry has made all health services free for pregnant women to improve access to care [36]. In 2016, the Ministry enabled midwives to perform immediate postpartum insertion of the LARC, in line with WHO guidelines [37]. This initiative aligns with WHO guidelines that recommend immediate postpartum contraception as a convenient and effective strategy to promote women’s health and delay subsequent pregnancies. This study is important because of the high rates of maternal mortality and morbidity, as well as the low prevalence of PLARC in Ethiopia [12]. Unwanted or closely spaced pregnancies contribute to most maternal deaths, and research has shown that short birth intervals increase the risk of maternal death, poor health, and poor pregnancy outcomes [38–40]. Therefore, preventing unwanted pregnancies and spacing births for 3–5 years are crucial components of safe motherhood.

However, the utilization of the PLARC was found to be significantly lower in Ethiopia, at only 14.2% [29]. Existing studies on this issue have focused on immediate postpartum contraceptive utilization in hospitals, neglecting women who have given birth at home or in other health facilities. Additionally, few studies have examined the prevalence and factors associated with PLARC in an extended postnatal period and at the community level [17, 31–33, 41]. Furthermore, these few studies have produced varied results and factors depending on the context [16, 19, 20, 26]. Therefore, determining the prevalence and identifying correlations with PLARCs in various contexts is important for designing appropriate guidelines to increase the coverage of PLARCs in specific settings. Therefore, it is important to determine the prevalence and identify correlations with PLARCs in different contexts to design appropriate guidelines and increase coverage in specific settings. This study aimed to assess the prevalence and determinants of PLARC in the extended postpartum period among married women of reproductive age in Shashogo District, Hadiya Zone, Central Ethiopia.

## Materials and methods

### Study design and area

This community-based cross-sectional study was conducted in Shashogo District, one of the seventeen districts in the Hadiya Zone, Central Ethiopia, from March 1, 2021, to April 15, 2021. The district is divided into thirty-four rural kebeles and two urban kebeles. It is bordered by the Siltie Zone to the east and north, the Halaba Zone and Kembata Tambaro Zone to the south, and the Lemo and Anlemo districts to the west. The district’s capital is Bonosha, which is situated 224 km away from Addis Ababa and 54 km from Hossana (the capital of the region). According to the population census projection by the Central Statistical Agency in 2019/20, the population of the Shashogo District is estimated to be 142,000, with males accounting for 70,574 and females accounting for 71,426. There were 4,913 pregnant mothers in the district. The district has one nonfunctional primary hospital, five health centers, and 36 health posts. Additionally, there are five high schools, 44 junior schools, and eight primary schools. The majority of the population relies on traditional agriculture, with maize being the main crop produced in the area.

### Population, inclusion criteria and exclusion criteria

The source population of this study consisted of all married women aged fifteen to forty-nine who had given birth in the district within the twelve months before the study period. The study population included randomly sampled women from this group. All married women who had given birth in the district within the twelve months before the study period were included in the study. However, married women with mental illness and those who were unable to hear or talk were excluded from the study because they were unable to provide the necessary information.

### Sample size determination and sampling procedures

#### Sample size calculation for objectives one and two

The sample sizes for both single and double population proportions were calculated using Epi Info version 7.1.

1. For a single population proportion, the following parameters were considered:

$P$= Proportion of PLARC = 25.4% [32].

*D* = margin of error = 0.05 with 95% confidence interval (CI)

*Z* = 1.96 (level of significance) and design effect = 2

*Nonresponse rate = 10*.

- The final sample included 640 mothers.

2. For the double population proportion, the following parameters were considered (Table 1):
1. P1  = % of outcomes among the exposed group
2. P2  = % of outcomes among the unexposed group
3. Confidence interval  =  95%, and power$=80\%$

**Table 1:** List of exposure variables used to calculate the sample size for factors associated with PLARC

Exposure variable	% of outcome among the unexposed group	% of outcome among the exposed group	Odds ratios (OR)	Sample** (n)	References
1. Education (Secondary)	**54.4**	**17.5**	**5.6**	**139**	[32]
2. Previous use of LARC(Yes)	59.2	28.6	3.6	207	[37]
3. Counseling on LARC during immediate postpartum period(Yes)	41.2	8.1	7.9	245	[32]
4. Discuss about LARC with partner (Yes)	64.7	9.7	17.0	66	[27]
5. Counseled on LARC during ANC (Yes)	50.7	16.4	5.2	154	[27]

** indicates samples with design effects 2 and nonresponse rates 10

According to the calculations of both objective sample sizes, it is apparent the first objective sample size calculation results in the largest sample size, which is 640. This figure was deemd an appropriate final sample size for the study conducted in the Shashogo district, Hadiya zone, Central Ethiopia, in 2021.

#### Sampling technique

To select the study subjects, a multistage stratified sampling technique was used. The subjects were categorized based on their place of residence, either rural or urban. The kebeles in the district were divided into urban and rural categories, and then one urban and nine rural kebeles were chosen using the lottery method. All households that included married women who had given birth within the 12 months preceding the data collection period were included in the study. The required data were collected from the relevant Kebele health post, which included the lists of households with women who had given birth within the 12 months prior to the commencement of data collection.

**Fig. 1:**
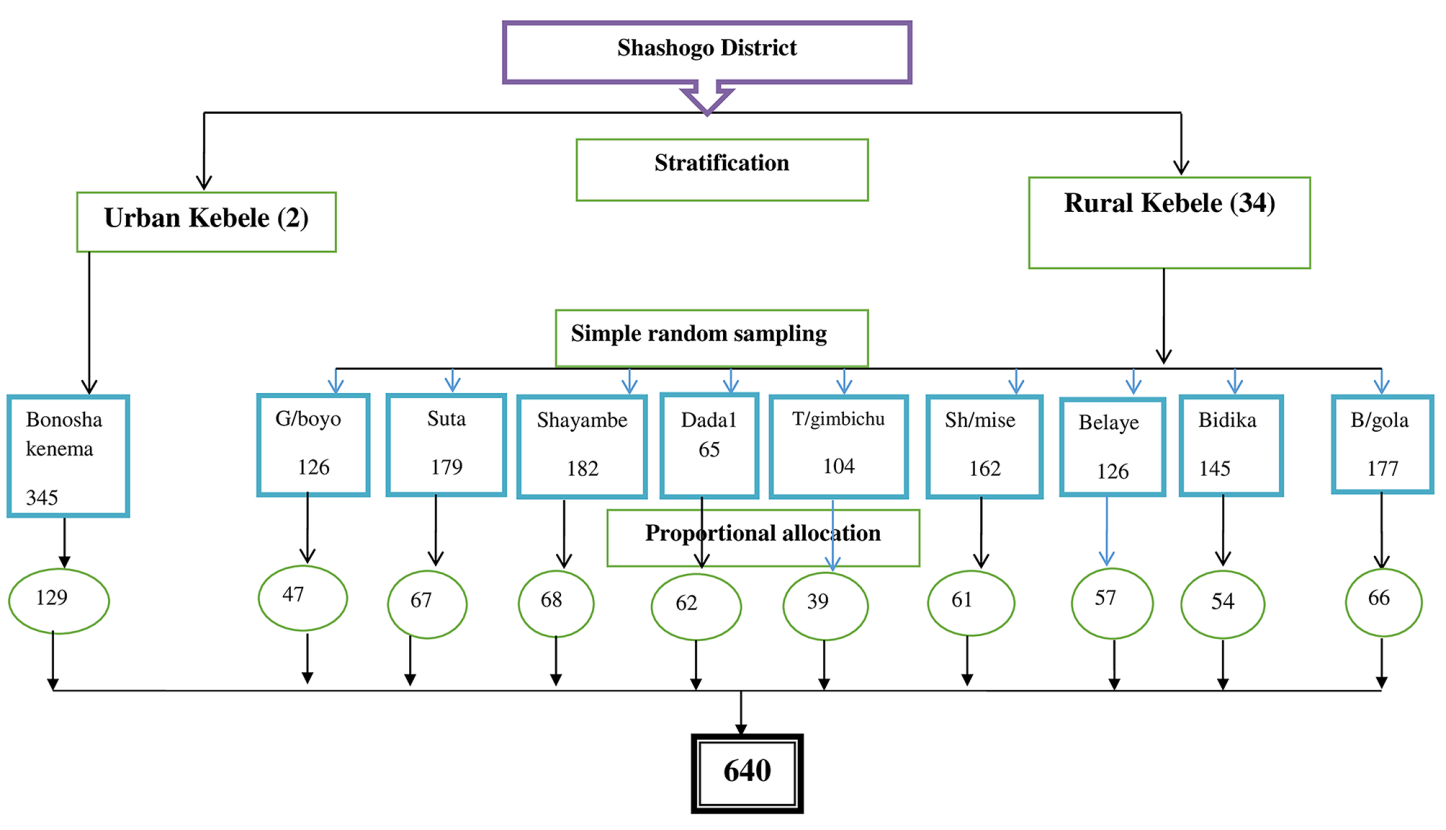
Sampling procedure for the study on prevalece and determinants of PLARC utilization among women who gave birth in the last 12 months prior to the study in Shashogo district, Central Ethiopia, 2021

The sample size was then allocated proportionally to each randomly selected kebele-based woman who met the criteria. Finally, a simple random sampling technique was used to select the study participants from each kebele (Fig. 1).

### Data collection procedure

The data were collected using an interviewer-administered, pretested, and structured questionnaire, which was adapted by reviewing the relevant literature and contextualizing it to the specific situation [19, 24, 30, 31, 41]The questionnaires aimed to gather information on sociodemographic factors, reproductive characteristics, knowledge, attitudes, and practices related to the PLARC. Four diploma nurses conducted the data collection under the supervision of two BSc-holder health professionals. To ensure data quality, the questionnaire (English version) was translated into Hadiyisa and then back into English by two different individuals to check for consistency. The content validity of the questionnaire was assessed by two assistant professors of maternity health nursing and one epidemiologist. Both the data collectors and supervisors received one day of training prior to the actual data collection, which covered the aim of the study, procedures, and methods for maintaining the confidentiality of the obtained information.

Two weeks prior to the commencement of actual data collection, a pretest of the questionnaire was conducted on a subset comprising 5% of the final sample. The principal investigator administered the pretest on the Ani-Lemo and subsequently transported it to the data collection site. In light of the pretest outcomes, we proceeded to enhance the content, adjust the structure, provide clearer instructions, and rectify any issues that were identified during the evaluation of the pretest. The questionnaire had a Cronbach’s alpha of 0.86 for knowledge-assessing tools and 0.8 for attitude-assessing tools. Supervision was provided throughout the data collection process.

### Operational definition

Utilization of the PLARC was defined as the use of any LAFPM (such as an IUD or subdermal hormonal implant) by women during the extended postpartum period. Knowledge about PPLAFPM was assessed through eight questions. Participants scoring ≥ 4 points were categorized as having good knowledge, while those scoring < 4 points were classified as having poor knowledge. Women’s attitudes towards LARCs were assessed using a five-point Likert scale with four items. Women who scored above the mean were considered to have a positive attitude, while those who scored below the mean were considered to have a negative attitude towards LARCs.

### Data processing and analysis

After completing the process of data collection, each questionnaire underwent a thorough manual review to ensure its completeness. The collected data were then encoded and entered into EpiData 3.1. Subsequently, the data were exported to SPSS version 25 for the purpose of conducting data validation, cleansing, and logistic regression analysis. To identify any missing values, frequency analysis was employed. Descriptive statistical analyses, including frequencies, means, and standard deviations for continuous variables, as well as percentages for categorical variables, were performed. Pearson’s chi-squared test was utilized to explore the relationship between each factor and the outcome variable. Finally, both bivariate and multivariable logistic regression analyses were conducted to assess the significant associations between the dependent and independent variables. Variables with a p value less than 0.25 in the bivariate analysis were entered into the multivariable logistic regression. Statistical significance was determined using a p value of less than 0.05, and the adjusted odds ratio (AOR) was reported with a 95% confidence interval (CI). Multicollinearity was assessed by examining the interaction between the independent variables using the variance inflation factor, which was found to be less than 5.

## Results

### Sociodemographic and reproductive characteristics of the participants

A total of 617 women in the reproductive age group participated, resulting in a response rate of 96.4%. The median age of the women was 28 years. More than two-thirds of the respondents (403, or 65%) identified as followers of Protestant Christianity, followed by Muslims (163, or 26.4%). In terms of educational status, 251 (40.7%) had no formal education. Three-fourths of the respondents (467, or 75.7%) resided in urban areas. Regarding occupation, more than three-fourths of the study participants (513, or 83.1%) were unemployed (Table 2).

### Reproductive characteristics and prevalence of PLARC

In this study, all participants had previously heard about LARCs. For those who had heard about them, the major source of information was health professionals; 360 (58.3%) and 363 (58.8%) women had ever used PLARC. This study revealed that the PLARC was used by 224 (36.3%) of the study participants. Additionally, the study revealed that 224 (36.3%) of the participants had utilized PLARC (Table 3).

### Knowledge and attitudes regarding LARC

In this study, two hundred fourteen (34.7%) respondents had good knowledge of PLARC. The findings showed that 560 (90.8%) of the participants responded that implants can prevent pregnancies for 3–5 years, and 450 (72.9%) responded that implants require minor surgery (Table 4). Three hundred nine (50.1%) respondents had a positive attitude toward LARCs. Among the 617 respondents, 367 (59.5%) disagreed and 44 (7.1%) strongly agreed that an implant can cause severe irregular bleeding. Three hundred sixty (58.3%) women disagreed, and 24 (3.9%) women strongly disagreed that insertion and removal of the implant is highly painful (Table 5).

**Table 2:** Sociodemographic characteristics of married women of reproductive age in Shashogo district, 2021

Variable	Categories	Number(*N* = 617)	%
Age in years	$\le$24	98	15.9
	25–29	301	48.8
	30–34	181	29.3
	35–49	37	6.0
Religion	Protestant	403	65%
	Catholic	25	4.1%
	Orthodox	26	4.2%
	Muslim	163	26.4%
Education	No education	251	40.7%
	Read and write	219	35.5%
	Primary school	103	16.7%
	Secondary and above	44	7.2%
Residence	Urban	150	24.3%
	Rural	467	75.7%
Occupation	Not-employed	513	83.1
	Employed	104	16.9
Average family monthly income (Ethiopian birr)	< 500	381	61.8%
	500–1000	120	19.4%
	> 1000	116	18.8%

### Determinants of PLARC utilization

The multivariable logistic regression analysis model identified several factors significantly associated with the utilization of PLARCs in the postpartum period. These factors included being aged 25–29 or 35–49 years, having a good level of knowledge about long-acting contraceptives, having a higher maternal education level, having a previous history of abortion, and receiving information about LARCs from healthcare workers. Compared to women with no formal education, women who attended primary education and secondary education and above were 3.3 and 3.5 times more likely to utilize LARCs in the postpartum period, respectively (AOR = 3.3; 95% CI: 1.6, 3.6) and (AOR = 3.5; 95% CI: 1.5, 8.3). Additionally, women with a good level of knowledge about LARCs were more than twice as likely to use them than were those with poor knowledge (AOR = 2.4; 95% CI: 1.5, 3.9). Furthermore, women with a history of abortion had a nearly threefold greater likelihood of using LARCs in the postpartum period than women without a history of abortion (AOR = 2.7; 95% CI: 1.3, 5.4). Finally, women who obtained information about LARCs from friends were 90% less likely to use them than were those who received information from healthcare workers (AOR = 0.1; 95% CI: 0.1, 0.2) (Table 6).

**Table 3:** Reproductive characteristics of married women of reproductive age in Shashogo district, 2021 (*n* = 617)

Variable	Categories	Number	%
Number of persons in the household	$<$ Three	70	11.4
	Four-six	266	43.1
	$\ge$Seven	281	45.5
Number of male children alive	$>$Three	554	89.8
	Four-six	53	8.6
	> seven	10	1.6
Number of female children alive	< three	519	84.1
	$\ge$four	98	15.9
Sex of a child wanted to have	Male	175	30.3%
	Female	40	6.9%
	No preference	136	23.5%
	Both equally	227	39.3%
A deciding body on the number of children	Wife	395	64%
	Husband	34	5.5%
	Together	188	30.5%
Discussing fertility issues with a husband	Yes	484	78.4%
	No	133	21.6%
Experience of abortion	Yes	69	11.2%
	No	548	88.8%
Source of Information about family planning method	Radio	63	10.2
	Friends	170	27.6
	Health workers	360	58.3
	Husband	24	3.9
Type of LARC ever used	Yes	363	58.8
	No	254	41.2
Current use of PLARC	Yes	224	36.3
	No	393	63.7
Type of PLARC currently using(*n* = 224)	Implants	192	85.7
	IUCD	32	14.3

**Table 4:** Knowledge of LARCs among married women of reproductive age in Shashogo district, 2021

Items (*n* = 617)	Options	Number	%
1. IUD prevent pregnancy for more than 10 years	No	259	42.0
	Yes	358	58.0
3. IUD is not appropriate for female at high risk of getting STDs	No	486	78.8
	Yes	131	21.2
5. IUD has no interference with sexual intercourse	No	401	65.0
	Yes	216	35.0
7. IUD is immediately reversible	No	412	66.8
	Yes	205	33.2
9. IUD cannot cause cancer	No	421	68.2
	Yes	196	31.8
11. Implant can prevent pregnancies for 3–5 years	No	57	9.2
	Yes	560	90.8
13. Implant require minor surgery	No	167	27.1
	Yes	450	72.9
15. Implant immediately reversible	No	182	29.5
	Yes	435	70.5
Over all Knowledge level toward LARC	Good	214	34.7
	Poor	403	65.3

**Table 5:** Attitudes toward LARC among married women of reproductive age in Shashogo district, 2021

Items	Strongly agree		Disagree		Neutral		Agree		Strongly disagree	
	N	%	N	%	N	%	N	%	N	%
1. Irregular bleeding due to using implant is severe	44	7.1	367	59.5	160	25.9	23	3.7	23	3.7
2. Insertion and removal of implant is highly pain full	24	3.9	360	58.3	94	15.2	100	16.2	39	6.3
3. Loosing privacy during IIUCD insertion is shame full	21	3.4	95	15.4	367	59.5	118	19.1	16	2.6
4. Using IUCD restricted from different work activity highly un acceptable	55	8.9	243	39.4	259	42	37	6	23	14.6
**Attitude toward PLARC**					Number			Percentage (%)		
	Positive attitude				309			50.1		
	Negative attitude				308			49.9		

## Discussion

Short-acting contraception is more widespread than LARC in resource-limited settings [14, 22]. The current study revealed that only 36.3% of women utilized PLARCs, which closely reflects real-world scenarios, particularly in developing nations. This significant finding underscores the urgent need for increased attention to the PLARC and the significant proportion of women who have limited family planning options.

The aforementioned statistic regarding the use of LARC during the postpartum period aligns with the results of two studies conducted in southern Ethiopia (36.5% and 36.7%) [19, 20] as well as a study conducted in primary healthcare facilities supported by the United States Agency for International Development Transform project in Amhara, Tigray, Oromia, and southern Ethiopia (36%) [42]. However, it is higher than the statistics reported in studies conducted in Arba-Minch (22.6%) [16], Hawassa city (25.4%) [28], Eastern Ethiopia (18.5%) [33], Uganda (8.5%) [25], and Tanzania (10.4%) [30]. This discrepancy can be attributed to variations in study design and participant characteristics. Previous research primarily used facility-based designs that focused on the immediate postpartum period when women were still under medical care. In contrast, this community-based study included women who delivered in hospitals, health centers, or at home. The discrepancies could also be due to differences in the characteristics of the participants across studies. For example, the Ugandan study exclusively involved rural women, which may have influenced the reported utilization rates compared to studies that included a more diverse population [25]. The discrepancies in utilization rates could also be influenced by geographical variations and cultural factors. Different regions or countries may have varying norms, beliefs, and access to healthcare services, which can impact the use of long-acting contraceptives. Studies focusing on different time periods postpartum may yield different utilization rates. Women’s attitudes and access to family planning methods may vary depending on how long it has been since they gave birth. The research findings highlight the low utilization of PLARCs, emphasizing the need for targeted interventions in low-income countries. Practical implications include implementing awareness programs, extending services to various delivery settings, and tailoring family planning initiatives to address regional disparities and diverse participant characteristics.

This study revealed that women aged 30–34 years exhibited lower utilization of PLARCs than women aged 25–29 or 35–49 years. This could be attributed to the former group needing more time to dedicate to educational pursuits and/or fulfill other obligations before considering another pregnancy. On the other hand, older women may already be occupied with care for their existing children and may require a gap between pregnancies to recover from the physiological changes associated with prior deliveries. They may also need additional time to attend to the needs of their current children instead of having more children. Additionally, older women may receive repeated information on modern contraceptives, including LARC, during antenatal care, family planning, and delivery. The experience gained from previous pregnancies may also contribute to increased utilization of PLARCs among older women. These findings align with studies conducted in Tanzania [30] and Ethiopia [43]. To address this issue, tailored educational programs and targeted counseling during antenatal care should be implemented to increase utilization rates, especially for this age group. Policy interventions should also consider the unique needs of women in different age categories to effectively promote the use of PLARCS.

The utilization of PLARCs was significantly associated with higher education levels. This finding suggests that education plays a crucial role in influencing the use of PLARCs. This conclusion aligns with research from Ethiopia [19, 31, 32], Uganda [25] and SSA [29]. This may be explained by the fact that women with higher education levels are more likely to have better access to information, critical thinking skills, and decision-making abilities. This enables them to understand the benefits of the PLARC and make informed choices about their reproductive health. Tailored contraceptive counseling should be provided to women with varying education levels to ensure that all individuals, regardless of their educational background, have the necessary information and support to make informed decisions about family planning. This emphasizes the importance of accessible and comprehensive reproductive health education for all women, regardless of their educational attainment, to empower them to make choices that align with their reproductive health objectives.

**Table 6:** Determinants of the utilization of PLARCs among married women in Shashogo district, 2021

Character	Category	PLARC use		OR at 95% CI	
		No	Yes	Crude OR	Adjusted OR
Residence	Urban	64	60	1.8(1.3,2.8)	1.4(0.8,2.5)
	Rural (ref.)	329	164	1	1
	$\le$24	56	42	2.7(1.6,4.6)	0.9(0.4,1.8)
	25–29	173	128	2.(1.8,4.1)	1.8(1.1,3.0) *
	30–34(ref.)	142	39	1	**1**
	35–49	22	15	2.4(1.2,5.2) *	8.7(3.6,21.5) **
Education	No education(ref.)	173	78	1	1
	Able to read and write	148	71	1.1(0.7,1.6)	0.9(0.5,1.5)
	Primary	53	50	2.1(1.3,3.3)	3.3(1.6,6.7) **
	Secondary and above	19	25	2.9(1.5,5.6)	3.5(1.5,8.3) **
Occupation	Not-employed(ref.)	320	193	1	1
	Employed	73	31	0.7(0.4,1) *	0.7(0.4,1.1)
Experience of abortion	Yes	55	14	2.4(1.3, 4.5) *	2.7(1.3, 5.4) **
	No (ref.)	338	210	1	1
Knowledge about LARC	Poor(ref.)	295	108	1	1
	Good	98	116	3.2(2.3,4.6) **	2.4(1.5,3.9) **
Source of information about LARC	Radio	39	24	0.7(0.4–1.2)	0.6(0.3,1.2)
	Friends	149	21	0.2(0.1–0.3) *	0.1(0.1, 0.2) **
	Health worker(ref.)	193	167	1	1
	Husband	12	12	1.2(0.5,2.4)	0.6(0.2,1.5)

Note 1: reference category, * p value < 0.05; AOR: adjusted odd ratio, COR: crude odd ratio***P$<$0.001

Furthermore, women who have a history of abortions are more likely to use PLARCs than are those who have never had an abortion. This can be attributed to the fact that women who have had abortions receive more opportunities for counseling and information on the ideal timing for conception following an abortion or childbirth. This knowledge aims to achieve an optimal birth interval. Women who possess a comprehensive understanding of postabortion or postdelivery contraceptive methods, as well as how to prevent unwanted pregnancies, are more likely to act upon this knowledge and engage in behaviors that mitigate associated risks. By providing focused support and education, healthcare providers can assist these women in making well-informed decisions to attain optimal birth intervals and avoid unintended pregnancies. This emphasizes the necessity for customized contraceptive counseling after abortion or delivery, empowering women to make choices that align with their reproductive health objectives. This finding is consistent with a study conducted in Uganda [25].

Moreover, women are more likely to access healthcare services when they have adequate information and guidance from healthcare professionals rather than when they rely on advice from peers who may have limited knowledge. This finding is supported by studies conducted in Ethiopia [16, 24, 28, 33, 42] and Tanzania [30]. Individuals who are well informed about healthcare services are more inclined to use them because they are better equipped to understand and assess the associated risks and benefits, both for their own health and the well-being of others. This conclusion can be explained by the fact that healthcare professionals have more experience and knowledge in counseling individuals on family planning methods, assisting women in their understanding, and facilitating the selection of the most effective options compared to friends or other sources of advice. Moreover, the reliance on healthcare professionals for guidance and information can lead to increased access to healthcare services, as individuals are better equipped to assess risks and benefits. This underscores the importance of tailored contraceptive counseling postabortion or delivery, which enables women to make informed decisions that support their reproductive health goals and overall well-being.

### Strengths and limitations of this study

The strengths of this study include the utilization of probability sampling to ensure the representation of the study participants, thus enhancing the reliability of the findings. Moreover, the implementation of various approaches to maintain data quality strengthens the credibility of the results. However, limitations exist. First, the scope of the study, which focused solely on married women, did not consider variables such as marital status, which could influence the use of postpartum long-acting contraceptives. Additionally, the absence of qualitative data limits the assessment of women’s perceptions and experiences related to the challenges associated with these contraceptive methods. Furthermore, recall bias may impact the accuracy of reported events, especially those that occurred further back in time from the data collection period. Participants may have difficulty accurately recalling past events or experiences, leading to inaccuracies in the data, such as family planning-related care (counseling during pregnancy and the immediate postpartum period) and previous contraceptive use. Finally, the potential influence of social desirability bias on participants’ responses is a concern for the validity of the findings. In this study, participants may feel pressured to report behaviors or attitudes that align with societal norms or expectations, such as using postpartum family planning methods.

## Conclusion and recommendations

The study findings suggest that a relatively small proportion of women utilize LARCs following childbirth, with Implanon being a more frequently selected option than IUDs. Various factors were found to be significantly associated with the use of long-acting contraceptives, including age, level of education, history of abortion, receipt of pregnancy counseling, and knowledge about long-acting contraceptives after childbirth. We propose that healthcare providers and policymakers concentrate on increasing awareness and knowledge regarding long-acting contraceptives among women of reproductive age, particularly those who have recently given birth. It is crucial to integrate contraceptive counseling and services into regular antenatal and postnatal care to ensure that all women have access to information and choices concerning postpartum contraception, thus enhancing acceptance. Tailored interventions for different age groups and educational backgrounds may also prove beneficial in promoting the use of long-acting contraceptives after childbirth. Further research and targeted interventions are necessary to address these barriers and improve access to these contraceptive methods for postpartum women.

## Data Availability

No datasets were generated or analysed during the current study.
